# An Integrated System for Detecting and Numbering Permanent and Deciduous Teeth Across Multiple Types of Dental X-Ray Images Based on YOLOv8

**DOI:** 10.3390/diagnostics15131693

**Published:** 2025-07-02

**Authors:** Ya-Yun Huang, Chiung-An Chen, Yi-Cheng Mao, Chih-Han Li, Bo-Wei Li, Tsung-Yi Chen, Wei-Chen Tu, Patricia Angela R. Abu

**Affiliations:** 1 Program on Semiconductor Manufacturing Technology Academy of Innovative Semiconductor and Sustainable Manufacturing, National Cheng Kung University, Tainan City 701401, Taiwan; m28124023@gs.ncku.edu.tw (Y.-Y.H.); wctu@gs.ncku.edu.tw (W.-C.T.); 2 Department of Electrical Engineering, Ming Chi University of Technology, New Taipei City 243303, Taiwan; 3 Department of General Dentistry, Chang Gung Memorial Hospital, Taoyuan City 33305, Taiwan; louiszzzzz@cgmh.org.tw; 4 Department of Electronic Engineering, Feng Chia University, Taichung City 40724, Taiwan; m1207569@o365.fcu.edu.tw (C.-H.L.); d1159017@o365.fcu.edu.tw (B.-W.L.); 5 Department of Electronic Engineering, National Cheng Kung University, Tainan City 701401, Taiwan; 6 Ateneo Laboratory for Intelligent Visual Environments, Department of Information Systems and Computer Science, Ateneo de Manila University, Quezon City 1108, Philippines; pabu@ateneo.edu

**Keywords:** dental X-ray images, periapical radiographs, bitewing radiographs, panoramic radiographs, permanent teeth, deciduous teeth, tooth numbering, YOLOv8, image preprocessing

## Abstract

**Background/Objectives:** In dental medicine, the integration of various types of X-ray images, such as periapical (PA), bitewing (BW), and panoramic (PANO) radiographs, is crucial for comprehensive oral health assessment. These complementary imaging modalities provide diverse diagnostic perspectives and support the early detection of oral diseases, thereby enhancing treatment outcomes. However, there is currently no existing system that integrates multiple types of dental X-rays for both adults and children to perform tooth localization and numbering. **Methods:** Therefore, this study aimed to propose a system based on YOLOv8 that integrates multiple dental X-ray images and automatically detects and numbers both permanent and deciduous teeth. Through image preprocessing, various types of dental X-ray images were standardized and enhanced to improve the recognition accuracy of individual teeth. **Results:** With the implementation of a novel image preprocessing method, the system achieved a detection precision of 98.16% for permanent and deciduous teeth, representing a 3% improvement over models without image enhancement. In addition, the system attained an average tooth numbering accuracy of 98.5% for permanent teeth and 96.3% for deciduous teeth, surpassing existing methods by 5.6%. **Conclusions:** These results might highlight the innovation of the proposed image processing method and show its practical value in assisting clinicians with accurate diagnosis of tooth loss and the identification of missing teeth, ultimately contributing to improved diagnosis and treatment in dental care.

## 1. Introduction

In dental medicine, the integration of various X-ray images was essential for a comprehensive assessment of a patient’s oral health. Each type of X-ray served a distinct diagnostic purpose. For instance, PA images were primarily utilized to evaluate the health of tooth roots and the surrounding bone tissue [1,2]. PANO images provided a broad view of the entire oral cavity, assisting practitioners in understanding the structure of all teeth and the jawbone [3,4,5]. BW images were mainly employed to detect cavities between teeth and assess occlusal relationships [6,7]. Through a thorough evaluation, practitioners gained a deeper understanding of a patient’s condition, thereby enhancing the chances of early detection of oral problems and improving treatment outcomes. The integration of multiple types of dental X-ray images not only aided in diagnosis but also supported the development of more effective treatment plans. Among these concerns, tooth loss was a significant global oral health issue closely linked to the progression of various oral diseases. According to the latest statistics, approximately 7% of individuals experienced tooth loss [8]. The consequences of tooth loss extended beyond physiological aspects, encompassing psychological and social challenges as well. Therefore, early prevention and treatment were vital for reducing the incidence of tooth loss.

The application of artificial intelligence (AI) in dentistry has garnered increasing attention, particularly in tooth localization. Numerous studies have utilized AI technology to automatically identify teeth [9,10,11,12,13], which not only enhances diagnostic accuracy but also accelerates the image processing workflow. In this study, the YOLOv8 model was employed for automatic localization due to its high accuracy, rapid processing speed, and strong adaptability to various image types, making it particularly suitable for processing dental imaging data [14,15]. The study integrated multiple types of dental X-ray images, performed image preprocessing and tooth numbering, and analyzed the incidence of tooth loss.

In dental image analysis, PA, BW, and PANO images were commonly used. However, these images often contained black areas that negatively impacted subsequent analysis and diagnostic accuracy. This study proposed a unified method to eliminate these black regions and standardize the images. To avoid distortion caused by stretching rectangular images into square formats, the study filled in the unused areas during the standardization process, thereby preserving image integrity and consistency. Subsequently, an image enhancement method and the YOLOv8 model were employed to identify the positions and numbers of teeth, as well as to detect tooth loss. This approach not only improved image quality but also enhanced the accuracy of tooth identification, providing more reliable data support for dental diagnostics. The main contributions of this study were as follows:

1. A unified system capable of handling a wide range of dental X-ray images.

Through an automated image standardization process, PANO, BW, and PA images with varying dimensions were normalized using cropping, resizing, and padding techniques. This preprocessing strategy significantly improved detection accuracy. The results showed that the proposed method enhanced model performance and provided practical clinical value by assisting clinicians in the accurate diagnosis of tooth loss and the identification of missing teeth.

2. The first system to support both adult and pediatric dental radiographs.

While most of the existing studies have focused solely on tooth localization and numbering in adult dental images, this study proposed an integrated approach capable of effectively handling the greater complexity of pediatric dental radiographs, particularly those containing both permanent and deciduous teeth. The system achieved superior accuracy and demonstrated high stability across different patient groups.

3. Improved performance in adult tooth localization compared to previous methods.

By incorporating image enhancement methods such as sharpening and median filtering, the clarity of tooth boundaries was significantly improved, enabling the YOLOv8 model to more accurately detect tooth shapes and positions. The experimental results showed that the proposed method achieved an accuracy of 98.5% in the localization and numbering of permanent teeth, representing a 5.6% improvement over the 93.28% accuracy reported in a previous study.

## 2. Methods

**Figure 1:**
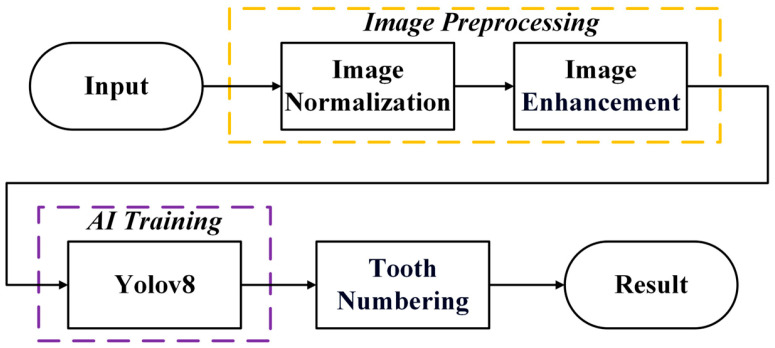
The workflow diagram for the localization and numbering of permanent and deciduous teeth using various types of X-rays with yellow cell indicating the image preprocessing step, and purple cell indicating the AI training step.

This study proposed an algorithmic workflow comprising image preprocessing, image enhancement, YOLOv8 model training, and tooth numbering, as shown in Figure 1. Initially, image preprocessing was performed to standardize image specifications, thereby preventing errors caused by inconsistencies during the training process. After training the YOLOv8 model, the coordinates corresponding to permanent and deciduous teeth were obtained. These coordinates were subsequently exported for accurate tooth numbering based on the Fédération Dentaire Internationale (FDI) tooth numbering.

### 2.1. Image Preprocessing

The image preprocessing was essential for improving the model’s accuracy and stability. Through effective preprocessing, images from various sources were standardized, ensuring compatibility across different types of dental X-ray images. This step also enhanced key dental features and reduced background noise, thereby minimizing its impact on the model’s performance. Such preprocessing was particularly important in this study, as the X-rays were obtained from diverse modalities and imaging conditions, making a well-designed preprocessing strategy essential for improving the detection performance of the YOLOv8 model. Two primary image preprocessing methods were employed in this study: image normalization and image enhancement.

#### 2.1.1. Image Normalization

**Figure 2:**
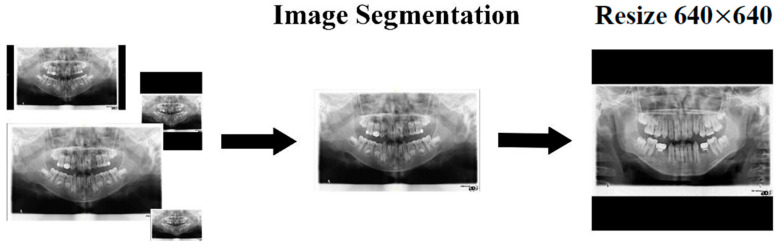
The flowchart of image normalization.

This study preprocessed commonly used dental X-ray images in clinical settings, including PANO, BW, and PA images. Given the variations in image size and tooth dimensions among these modalities, preprocessing was a crucial step. The original Digital Imaging and Communications in Medicine (DICOM) files were converted into single-channel grayscale images to reduce computational complexity while preserving essential anatomical details, as shown in Equation (1). The grayscale images were cropped to eliminate excess black borders and resized to meet the fixed input size required by the model. To avoid tooth distortion from direct resizing, the images were scaled while maintaining their original aspect ratios. Specifically, the longest side of each image was adjusted to 640 pixels, and the remaining areas were padded accordingly. This normalization approach ensured that the images remained distortion-free while being standardized to a uniform size of 640×640 pixels, as shown in Figure 2. This process enabled the YOLOv8 model to effectively handle all types of dental X-ray images, thereby improving accuracy in both tooth localization and numbering.

(1) Gray=0.299×Red+0.587×Green+0.114×Blue

#### 2.1.2. Image Enhancement

**Figure 3:**
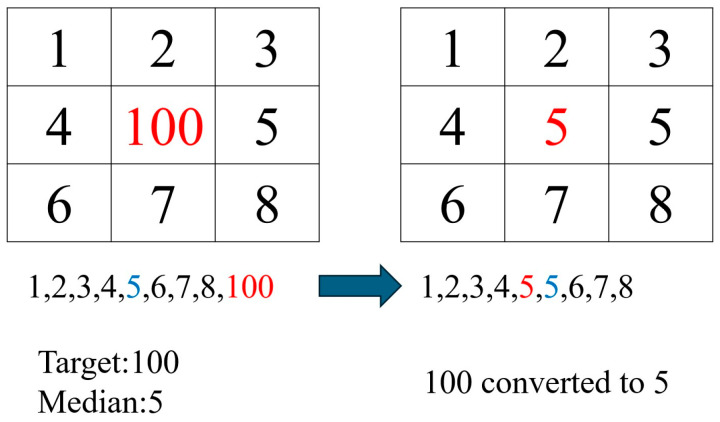
The schematic diagram of the principle of median filtering with red text indicating the center pixel and blue text indicating the sorted median.

**Figure 4:**
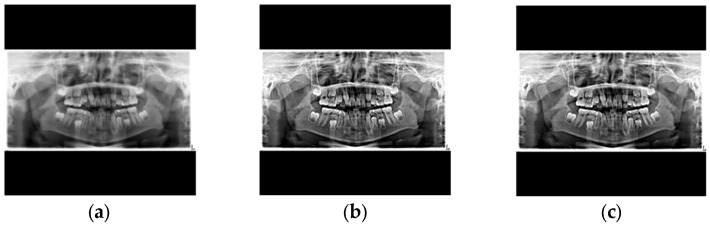
The results of image enhancement: (**a**) original image, (**b**) sharpening, (**c**) sharpening and median filtering.

When processing dental X-rays, the small gaps between teeth posed a significant challenge for deep learning models in accurately identifying individual teeth, particularly in PANO images. To address this problem, the study emphasized tooth contours through image enhancement during the preprocessing stage. This enhancement method significantly improved the model’s identification accuracy, providing a solid foundation for subsequent tooth analysis. Sharpening [16,17] was employed to enhance tooth edges and highlight their contours, as defined in Equation (2). However, sharpening also introduced additional noise into the images. To mitigate this effect, median filtering [18,19] was applied after sharpening. The principle of median filtering involved examining each pixel, comparing it with its surrounding neighbors, and replacing the original pixel value with the median of the sorted grayscale values, as shown in Figure 3. This method effectively removed noise while preserving the contour features of the teeth. A comparison of images before and after enhancement is presented in Figure 4.

(2) $$\nabla^{2}f=\frac{\partial^{2}f}{\partial x^{2}}+\frac{\partial^{2}f}{\partial y^{2}}$$

### 2.2. Tooth Localization and Numbering System

#### 2.2.1. Build Database

**Figure 5:**
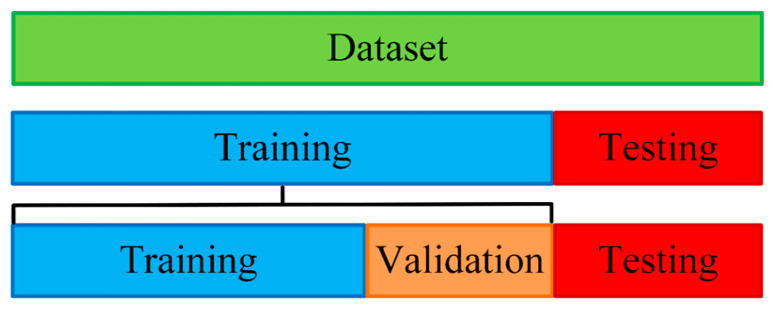
The partitioning of training data for the model.

During the dataset construction phase for training the YOLOv8 model [20], this study implemented a detailed partitioning and management strategy to enhance the effectiveness of model training, as shown in Figure 5. The overall dataset was initially divided into two main components. Initially, 80% of the dataset was allocated for training and 20% for testing. The training set was then further segmented, where 70% was allocated to training and 30% to validation. This partitioning method allowed the model to learn features from a substantial portion of the data while using the validation set for real-time evaluation and hyperparameter tuning, thereby reducing the risk of overfitting.

#### 2.2.2. Yolov8 Model

**Figure 6:**
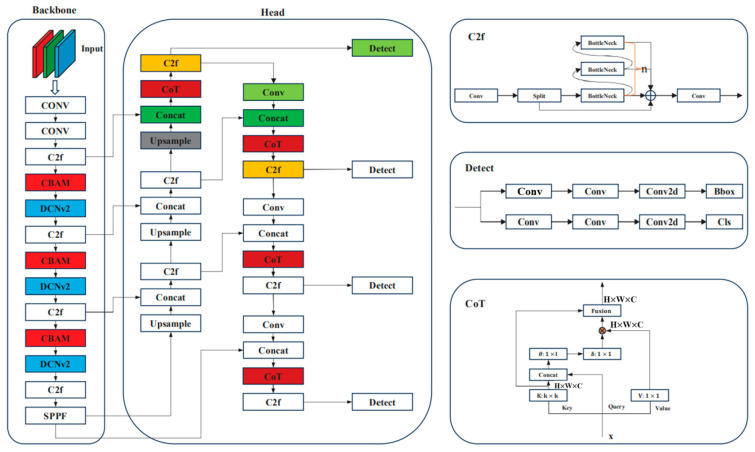
YOLOv8 model architecture diagram [23].

To accurately localize both permanent and deciduous teeth in various types of dental X-ray images, YOLOv8 was adopted. YOLOv8 is an advanced deep learning-based object detection model known for its real-time performance, multi-scale detection capability, support for multiple object instances, and high accuracy [21,22]. In this study, the hardware and software resources used are summarized in Table 1. These resources provided sufficient computational power and a robust development environment, ensuring that the YOLOv8 model was trained and tested efficiently, resulting in accurate detection outcomes. The architecture of the YOLOv8 model is shown in Figure 6.

#### 2.2.3. Hyperparameter

In YOLOv8, configuring hyperparameters was essential for both training and detection tasks. Hyperparameters are predefined settings that must be established prior to training, and they have a significant impact on the learning process and overall model performance. By fine-tuning these hyperparameters, this study successfully optimized the training process and improved the model’s accuracy. The specific hyperparameter settings used in this study are summarized in Table 2.

### 2.3. Tooth Numbering

**Figure 7:**
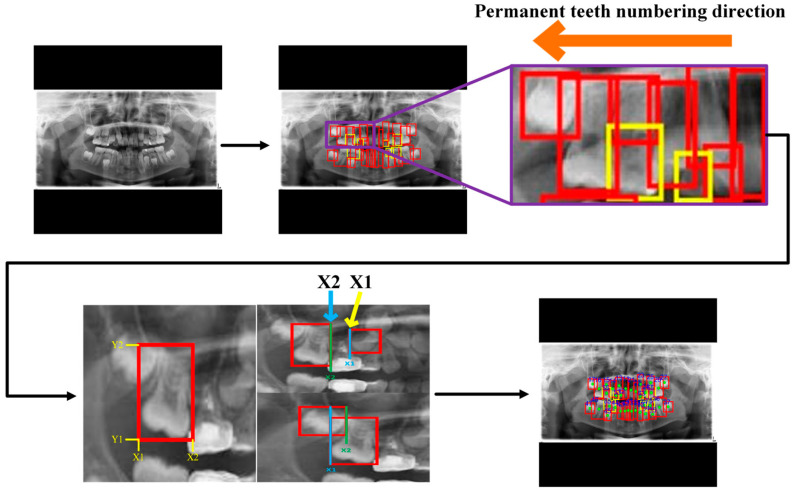
The method for tooth localization in PANO image in this study.

In PANO images, this study assigned an international identification code to each tooth following the FDI World Dental Federation notation system [24,25]. This systematic numbering enabled dentists to accurately identify teeth and track anatomical structures or abnormalities, thereby enhancing diagnostic precision and treatment planning. After obtaining the tooth localization coordinates from the YOLOv8 model, these coordinates were used to determine the central point of the occlusal surface, which was used to divide the dentition into four quadrants. Tooth numbering was performed using the quadrant-based method, starting from the upper left and proceeding clockwise. For permanent teeth, the first digit ranged from 1 to 4, and for deciduous teeth, from 5 to 8. The second digit for permanent teeth was assigned sequentially from the midline outward. In cases of missing teeth, the number of gaps was estimated based on the X1 and X2  coordinates, and the missing tooth number was determined by matching the number of missing teeth with the gaps, as shown in Figure 7.

**Figure 8:**
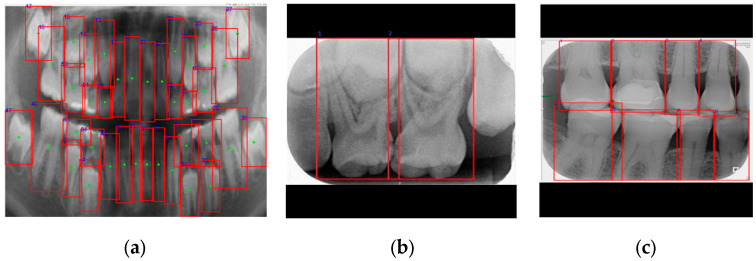
The results of tooth numbering images for various image types in this study. (**a**) The PANO image. (**b**) The PA image. (**c**) The BW image.

For deciduous teeth, the second digit was assigned based on their distance from the nearest permanent tooth along the X-axis. The numbering process was relatively straightforward in PA and BW images; however, it remained a critical step. In PA images, teeth were numbered from left to right, while in BW images, numbering followed a left-to-right, top-to-bottom sequence, as shown in Figure 8. Although the numbering method adopted in this study was simple, it ensured consistent processing and accurate labeling across different image types. This consistency enhanced the model’s diagnostic and localization performance across various imaging formats. Moreover, the standardized numbering system enabled the unification of data from multiple image sources, thereby improving the generalization and practical applicability of the proposed automated dental diagnostic model.

## 3. Results

This study proposed an automated system for detecting and numbering both permanent and deciduous teeth across various types of dental X-ray images. To maintain clinical relevance, all radiographic images used in this study, including PA, BW, and PANO, were sourced from the imaging database of Chang Gung Memorial Hospital in Taoyuan, Taiwan. The images were anonymized by certified dentists and randomly selected to ensure unbiased sampling. The study protocol received approval from the Institutional Review Board (IRB) of Chang Gung Memorial Hospital (IRB No. 202400084B0). Tooth annotations and condition labeling were performed by an attending dentist with more than three years of clinical experience.

To ensure a fair and objective evaluation of the proposed method, five performance metrics were utilized: Accuracy, Precision, Recall, F1-Score, Average Precision (AP), and mean Average Precision (mAP). The definitions of these metrics are provided in Equations (3)–(8), and Table 3 presents a schematic representation of the confusion matrix. Notably, for all the metrics, higher values indicate better model performance.

(3) $$\mathrm{Accuracy}=\frac{\mathrm{TP}+\mathrm{TN}}{\mathrm{TP}+\mathrm{TN}+\mathrm{FP}+\mathrm{FN}}$$

(4) $$\mathrm{Precision}=\frac{\mathrm{TP}}{\mathrm{TP}+\mathrm{FP}}$$

(5) $$\mathrm{Recall}=\frac{\mathrm{TP}}{\mathrm{TP}+\mathrm{FN}}$$

(6) $$F1-\mathrm{Score}=2\times \frac{\mathrm{Precision}\times \mathrm{Recall}}{\mathrm{Precision}+\mathrm{Recall}}$$

(7) $$AP=\int_{0}^{1}{Precision}_{\left(\mathrm{Recall}\right)}d\mathrm{Recall}$$

(8) $$mAP=\frac{1}{n}\sum_{i=0}^{n}{AP}_{i}$$

**Figure 9:**
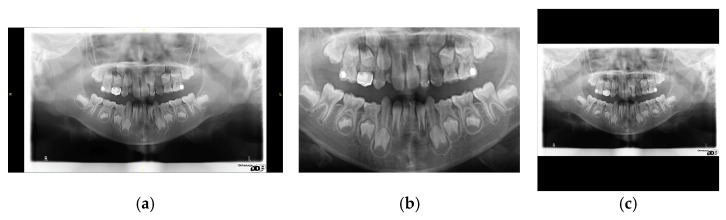
The results of image normalization using three methods: (**a**) resize of the original image, (**b**) resize after cropping, (**c**) resize after cropping with added padding.

This study evaluated three image normalization methods to enhance the performance of the YOLOv8 model in localizing and numbering teeth across various types of dental X-ray images, as shown in Figure 9. The first method followed the commonly used approach in previous studies, where the original image was scaled directly to a uniform size [26]. Although this approach simplified the model training process, it proved less effective in preserving critical dental features and achieving high detection accuracy. In contrast, this study proposed two alternative normalization methods to optimize model performance. The first method involved cropping and resizing the image with additional padding, while the second method removed unnecessary black borders before resizing the image. The results indicated that the latter method, removing black borders prior to resizing, achieved the best performance. This method not only maintained higher detection accuracy across different types of dental images but also better preserved anatomical detail. The effectiveness of the proposed method was validated by comparing it with the conventional normalization approach described in [26]. As shown in Table 4, the proposed normalization method achieved a recall rate of 99.22% for permanent teeth and 96.5% for deciduous teeth in PANO images. Compared to the baseline method, the study showed consistent improvements in detecting both permanent and deciduous teeth across multiple types of dental radiographs. These results confirmed that the proposed preprocessing strategy significantly enhanced the model’s ability to accurately identify teeth. By applying padding more effectively and preserving image integrity, the method substantially improved overall detection performance.

By reinforcing the edges and structural details of teeth within the images, the enhancement process enabled the YOLOv8 model to achieve higher precision in tooth localization. Following enhancement, the model’s detection performance in PANO images showed significant improvement, as presented in Table 5. Specifically, the precision for permanent teeth increased by 1.3%, while the precision for deciduous teeth improved by 4.8%. These results show that image enhancement not only clarified key dental features but also substantially improved the performance of the model for tooth numbering. This enhancement provides a more reliable foundation for clinical application.

This study also evaluated more recent models, including YOLOv5, YOLOv11, and Faster R-CNN for comparison. The results are shown in Table 6. It was found that the performance of YOLOv5 was comparable to that of YOLOv8, whereas Faster R-CNN showed noticeably inferior results. It is worth noting that YOLOv11 achieved the highest accuracy among all the tested models. However, YOLOv11 required significantly greater computational resources and longer training time compared to YOLOv8 due to its larger model size. Therefore, considering the trade-off between performance and efficiency, YOLOv8 was ultimately selected as the primary model for this study.

**Figure 10:**
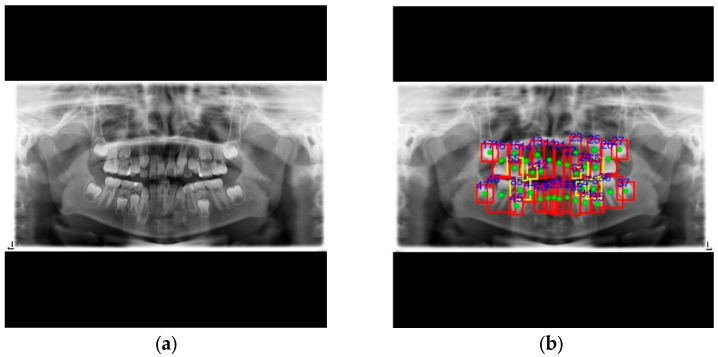
The example of PANO image analysis: (**a**) original image, (**b**) detection results with the red block indicates the tooth localization, and the number represents the tooth number.

**Figure 11:**
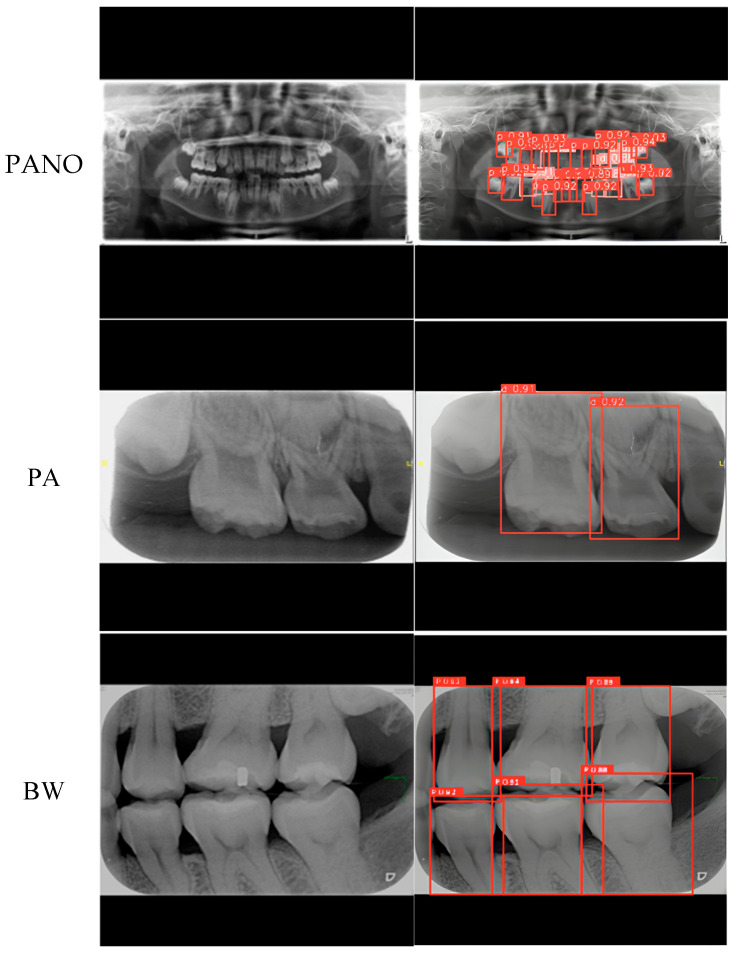
The results of the localization of various dental X-ray images in this study.

This study tested a PANO image containing both permanent and deciduous teeth. The identification results obtained using the proposed method are shown in Figure 10 and Table 7. In Table 7, the model’s predictions were compared with manual annotations by a dentist, where ‘0’ indicates absence and ‘1’ indicates presence. The results show consistent performance between the proposed method and manual labeling. The proposed system effectively assists dentists in the clinical localization and annotation of teeth across various types of dental X-ray images. Examples of detection results for three commonly used X-ray modalities are shown in Figure 11.

This study proposed an innovative algorithm for numbering both permanent and deciduous teeth in pediatric dental X-ray images. By advanced image enhancement and localization methods, the proposed algorithm in this study effectively identified the position of each tooth and assigned the corresponding tooth number. Compared to traditional methods, the algorithm showed superior capability in handling complex pediatric dental images, particularly those containing mixed dentition, and showed higher accuracy and stability. In terms of numbering accuracy, the proposed method significantly outperformed previous approaches. While a previous study reported an overall accuracy of 93.28% for numbering permanent teeth using a conventional algorithm [24], the method in this study achieved an overall accuracy of 98.50% for permanent teeth, as shown in Table 8. Additionally, this study attained an average accuracy of 96.3% for numbering both permanent and deciduous teeth, and an accuracy of 88.6% for detecting missing teeth, as presented in Table 9.

## 4. Discussion

This study presented an automated system that integrates various types of dental X-ray images for accurate localization and numbering of both permanent and deciduous teeth. The results demonstrated the effectiveness of the YOLOv8 model in dental radiograph analysis, particularly in tooth localization and numbering tasks. To address variations in image characteristics, a normalization method was developed for three types of dental X-ray images. Compared to the conventional approach of resizing images to a fixed dimension [26], the proposed normalization method yielded consistent improvements across all image types. Furthermore, image enhancement techniques were introduced to further improve model accuracy. The results showed enhanced recognition performance for both permanent and deciduous teeth, indicating that noise removal has a positive impact on model effectiveness. Compared to the method in [24], the proposed approach achieved a 5.6% increase in accuracy for permanent tooth localization and numbering.

This achievement underscores the efficacy of the method employed in image processing and tooth numbering, thereby assisting clinicians in diagnosing tooth loss with greater precision. By integrating various types of dental X-ray images, the study not only improves the accuracy of tooth detection but also facilitates the timely identification of missing teeth. Although the model showed strong performance across different image modalities, there remains potential for further improvement. For instance, addressing image noise and analyzing its impact on model performance may contribute to future enhancements. Moreover, future studies should explore the incorporation of additional clinical conditions and assess the model’s applicability across a broader range of clinical scenarios to further confirm its robustness and clinical utility.

## 5. Conclusions

This study proposed the YOLOv8 model for the automatic localization and counting of teeth in various types of dental X-ray images, significantly improving the detection accuracy for both permanent and deciduous teeth. Through the proposed image preprocessing and enhancement methods, the model exhibited robust performance in dental radiograph analysis, offering an efficient and accurate diagnostic tool for clinical dentistry. The proposed system enables clinicians to efficiently detect missing teeth, supporting the formulation of more effective treatment plans. The findings of this study not only underscore the potential of artificial intelligence in dental image analysis but also establish a foundation for future automated diagnostic systems. Subsequent studies should explore the integration of diverse imaging modalities for disease detection and enhance the model’s applicability in more complex clinical scenarios.

## Figures and Tables

**Table 1:** Hardware and software equipment applications.

Hardware Platform	Version
CPU	AMD Ryzen 7 5700X 8-Core Processor
GPU	NVIDLA GeForce RTX3060 Ti
RAM	32G
Software Platform	Version
OS	Window 11 Pro
Python	3.11.4

**Table 2:** YOLOv8 hyperparameter configuration.

Hyperparameters	Value
Optimizer	Adam
Image Size	640
Initial Learning Rate	0.005
Epoch	500
Min Batch Size	16

**Table 3:** An example of the confusion matrix.

	True	Positive	Negative
Predicted			
Positive		TP	FP
Negative		FN	TN

**Table 4:** The comparison for the normalization of various dental X-ray images.

		Method in [26]		This Work	
	Teeth	Permanent	Deciduous	Permanent	Deciduous
PANO	Precision ↑	95.90%	89.69%	97.01%	93.13%
	Recall ↑	98.65%	94.27%	99.22%	96.50%
PA	Precision ↑		79.44%		83.80%
	Recall ↑		95.00%		95.37%
BW	Precision ↑	95.21%		96.83%	
	Recall ↑	99.24%		99.56%	

The upward arrow indicates that higher values are preferred.

**Table 5:** The results of enhancement methods for CNN identification.

	Cutting Black Borders + Padding	Sharpening + Median Filtering
Precision ↑	95.07	98.16
Recall ↑	97.86	98.44
mAP50 ↑	98.22	98.48
mAP50~95 ↑	70.18	72.94
F1-score ↑	96.44	98.30
Image	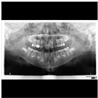	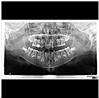

The upward arrow indicates that higher values are preferred.

**Table 6:** The comparison of multiple models.

		YOLOv5		YOLOv11		Faster R-CNN		YOLOv8	
	Teeth	Permanent	Deciduous	Permanent	Deciduous	Permanent	Deciduous	Permanent	Deciduous
PANO	Precision ↑	96.2%	88.7%	98.3%	95.7%	70.8%	49.6%	97.0%	93.1%
	Recall ↑	98.6%	93.5%	99.4%	97.8%	65.3%	28.1%	99.2%	96.5%
PA	Precision ↑		96.5%		99.5%				83.8%
	Recall ↑		92.6%		95.6%				95.4%
BW	Precision ↑	95.7%		99.0%				96.8%	
	Recall ↑	98.5%		99.8%				99.6%	
Training Time (Min)		10		47		157		9	
Model Size (MB)		7.5		50		170		6	

The upward arrow indicates that higher values are preferred.

**Table 7:** The comparison of results for this work and manual labeling.

		11	12	13	14	15	16	17	18	21	22	23	24	25	26	27	28
Permanent	Manual labeling	1	1	1	1	1	1	1	0	1	1	1	1	1	1	1	0
	This work	1	1	1	1	1	1	1	0	1	1	1	1	1	1	1	0
		31	32	33	34	35	36	37	38	41	42	43	44	45	46	47	48
	Manual labeling	1	1	1	1	1	1	1	0	1	1	1	1	1	1	1	0
	This work	1	1	1	1	1	1	1	0	1	1	1	1	1	1	1	0
Deciduous		51	52	53	54	55				61	62	63	64	65			
	Manual labeling	0	0	1	0	1				0	0	1	0	1			
	This work	0	0	1	0	1				0	0	1	0	1			
		71	72	73	74	75				81	82	83	84	85			
	Manual labeling	0	0	0	1	1				0	0	0	0	1			
	This work	0	0	0	1	1				0	0	0	0	1			

**Table 8:** The comparison between different proposed methods.

	11	12	13	14	15	16	17
Method in [24]	99.1	98.2	97.3	96.4	95.5	94.6	92.9
This Work	100	98.9	98.9	98.9	96.7	98.9	97.8
	21	22	23	24	25	26	27
Method in [24]	97.3	96.4	93.8	91.1	87.5	85.7	96.6
This Work	100	98.9	98.9	100	98.9	97.8	96.7
	31	32	33	34	35	36	37
Method in [24]	98.2	96.4	98.2	95.5	94.6	92.9	92.0
This Work	100	97.8	100	94.4	97.8	100	100
	41	42	43	44	45	46	47
Method in [24]	99.1	97.3	92.9	91.1	87.5	86.6	84.8
This Work	100	95.6	97.8	96.7	98.9	100	100

**Table 9:** The identified results for this study.

FDI Tooth Number	Accuracy ↑
11, 21, 24, 31, 33, 36, 37, 41, 46, 47	100%
12, 13, 14, 16, 22, 23, 25, 45, 81	98.9%
17, 26, 32, 35, 43, 61, 71	97.8%
15, 27, 44, 51	96.7%
42	95.6%
34, 55, 64, 74	94.4%
54, 73, 82	93.3%
52, 62, 63	92.2%
65, 72, 83, 85	91.1%
53	90.0%
75	88.9%
84	87.7%

The upward arrow indicates that higher values are preferred.

## Data Availability

The data presented in this study are available on request from the corresponding author. The data are not publicly available due to privacy or ethical restrictions.
